# PEAS: An Application for Autonomous Precision Conformation Sampling

**DOI:** 10.1021/acs.jcim.5c02104

**Published:** 2025-11-05

**Authors:** Mithony Keng, Kenneth M. Merz

**Affiliations:** † Department of Chemistry, 3078 Michigan State University, East Lansing, Michigan 48824, United States; ‡ Department of Biochemistry and Molecular Biology, Michigan State University, East Lansing, Michigan 48824, United States

## Abstract

Molecular modeling tools are routinely utilized in computational chemistry and computational biology projects. The ongoing advancements in hardware and software have made modeling diverse chemical systems more accurate and computationally affordable. However, with many software tools available to perform multiple relevant tasks, selecting the best workflow can become daunting in itself. In one of our recent works, we developed a workflow to assign chemical structures to experimental ion mobility mass spectrometry collisional cross-section (CCS) values. This requires multiple steps, including protonation state assignment, relevant conformational search, and conformation similarity filtering, to deliver a manageable workload for downstream quantum mechanical (QM) calculations. To simplify running our workflow, we have developed an open-source, user-friendly Python application called PEAS (**p**recise **e**nsemble **a**utonomous **s**ampling) that effectively streamlines the result chain through vertical modeling engine integration to limit user intervention. Since the crucial steps prior to quantum mechanical processing in modeling are charge state determination and relevant conformation sampling, we have therefore incorporated SEER (charge state predictor), Confab (conformation generator), and CCS Focusing (conformer filtering) into the development of PEAS. These engines have been separately validated and confirmed for efficiency and acceptable accuracy, and thus, we report that their unified performance also delivers similar outcomes. Documentation for PEAS and its Google Colab executable platform is available at https://github.com/mitkeng/peas.

## Graphical abstract

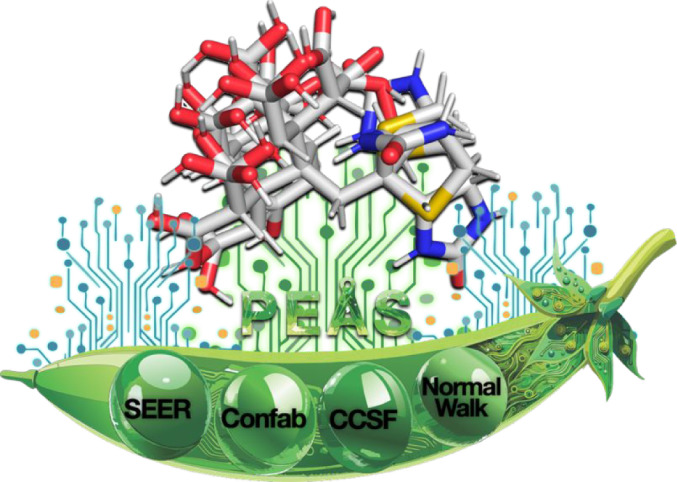

## Introduction

In computational chemistry and biology, insights into the structure and interaction of molecules are important for a deeper understanding of experimental observables. Chemical structure assignment is especially valuable in gas-phase-based experiments like mass spectrometry, where only information regarding molecular mass and/or size is obtained as an output. Specifically, computational tools can greatly enhance typical ion-mobility mass spectrometry (IM-MS) experiments, in which molecular mass and size are derived from the resulting collision cross-section (CCS) data. Since CCS is essentially an emergent property of IM-MS parameters (e.g., adduct charge state, conformation, and collision gas), experimentally relevant ground-state geometries can be computationally derived successfully with the proper recovery of these parameters during the ion-neutral collision simulation for theoretical CCS calculation.

To generate a theoretical CCS—which depends on a gas-phase equilibrium geometry and atomic partial charges—that aligns well with experimental data, accurate charge state assignment and relevant conformational space sampling are prerequisites. Historically,^1–4^ the steps that we have depended on to produce valid chemical structures with acceptable agreement with experimental CCS values (i.e., CCS error ≤3%) are shown in Figure 1. After the conformation generation step (see Figure 1), the subsequent steps for structure assignment involve charge state ensemble sampling, conformer similarity reduction, quantum mechanical optimization and single-point energy calculation, CCS calculation, and relative energy scoring. Lastly, the energy-minimum candidate structures are evaluated for experimental validity by comparing calculated CCS with the experimental reference (when available). For most CCS references relevant to this work, the experimental source is the drift tube ion mobility mass spectrometry (DTIMS) operating at a low, uniform electric field with nitrogen as the buffer gas. Furthermore, experimental DTIMS CCS values are directly calculated from the ions’ arrival time data using the Mason-Schamp equation for low-field mobility.

**Figure 1:**
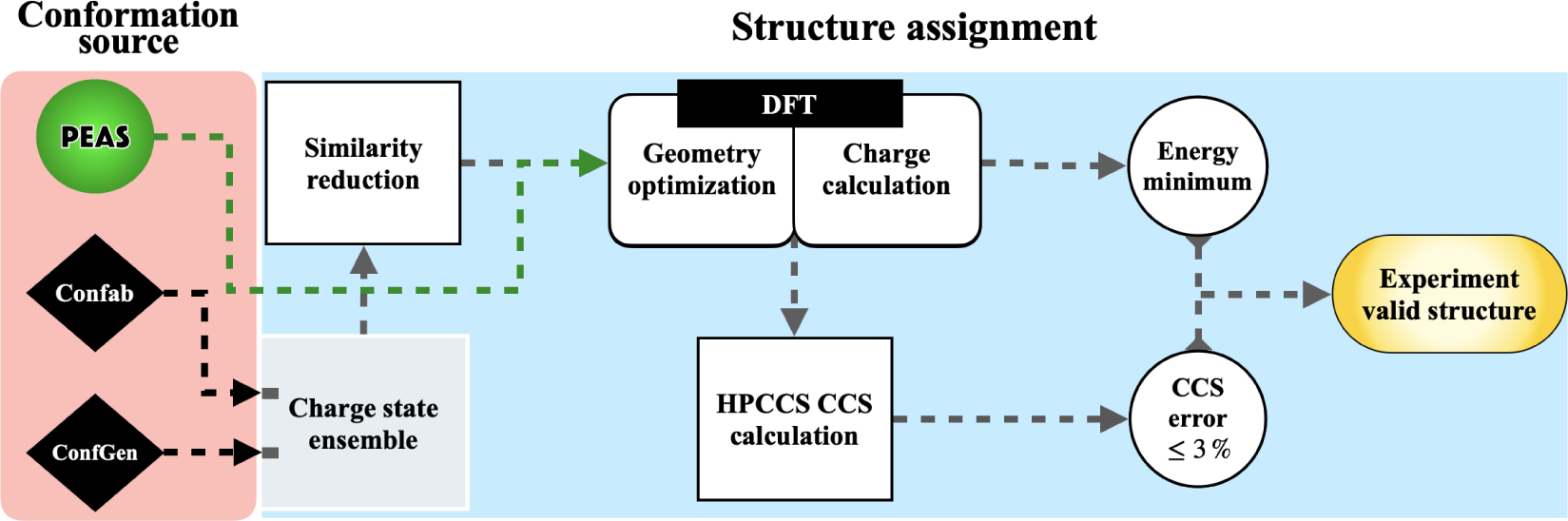
Workflow scheme for producing an experimentally viable gas-phase structure. The standard part of the workflow (blue segment) used to assign IM-MS-relevant structures consists of clustering, QM-level geometry optimization and partial charge calculation, CCS calculation, relative energy scoring, and CCS error evaluation. The variable part of the workflow (red segment) is the conformation sourcing. PEAS (green arrow) autonomously completes the modeling steps up until the QM (i.e., DFT) step. For validation and benchmarking, the conformation source performance is quality-assessed using the steps within the standard part.

Currently available modeling packages like POMICS,^4^ CREST,^5^ Auto3D,^6^ and ISiCLE^7^ offer automated and relatively efficient paths for generating low-energy gas-phase conformations for tautomeric or protomeric molecular species. The resulting candidate conformers can then be used as precursors for downstream quantum mechanical processing to assign equilibrium structures from IM-MS-derived CCS values. In prior work,^8^ we implemented POMICS within the Snakemake software framework to reduce the need for user intervention between programmatic steps and, thus, improve the results turnaround time. The platform for the aforementioned software packages, however, is not readily executable or easily operated by users with very limited or no computational modeling software experience.

More recently, we have developed two new software engines called SEER^9^ (**s**tate **e**nsemble **e**nergy **r**ecognition) and CCSF^3^ (**c**ollision **c**ross-**s**ection **f**ocusing) for accurate protonation charge state assignment and for experimentally relevant conformation filtering, respectively. Prior to vertically integrating SEER into a single platform, we used Dimorphite-DL^10^ or Epik^11^ (both p*K*_a_-based approaches) in combination with quantum mechanical (i.e., density functional theory) energy scoring (via Gaussian 16 software^12^ or QUICK^13^) to obtain experimentally viable gas-phase charge states for subsequent conformation sampling. Although sampling all protonation/deprotonation models with QM is an option to improve on p*K*_a_ approaches, it becomes computationally impractical when systems possess numerous titratable sites. Thus, since SEER’s assigned charge states align well with those determined by DFT, we can reduce the charge state sampling volume and iterative dependence on the costly QM step for IM-MS-relevant charge state assignment. With CCSF, we made it possible and practical to prescreen for IM-MS experimentally viable conformers after conformation generation, which helps to eliminate imprecise conformation sampling and prevent the occurrence of modeling jobs that may not yield viable results with respect to an experimental reference. Simply put, both SEER and CCSF have been demonstrated to effectively streamline and reduce the work volume that enters the rate-limiting quantum mechanical step (e.g., DFT calculations).

For conformation generation, rule- or knowledge-based generators like ConfGen,^14^ ETKDG,^15^ OMEGA,^16^ and FROG2 ^17^ are available through open-source or closed-source platforms. Historically, we have routinely used ConfGen and ETKDG; however, we found that Confab^18^ from Open Babel offers the best compromise between availability and performance. In particular, Confab performance differentiators include open-source access, low-energy conformer generation combined with structure similarity filtering, and Python notebook compatibility. By integrating Confab with SEER and CCSF under a single platform, we can perform all necessary modeling functions up to the quantum mechanical (QM) step.

Here, we introduce PEAS (**p**recise **e**nsemble **a**utonomous **s**ampling), a multimodel Python application with a user-friendly interface to streamline the generation of conformation ensembles. With a user-initiated chemical input, PEAS implements the following programmatic sequence: 1) SEER for assigning equilibrium charge state, 2) Confab for conformation generation, 3) CCSF for conformational space screening, and 4) normal walk to reduce redundant conformations (see Figure 2). The resulting output ensemble is expected to be refined by relative energy, structure dissimilarity, and, ultimately, experimental relevance, which, in consequence, should help to enhance the success rate of the modeling effort. Additionally, PEAS delivers preliminary results that enable a user to have some degree of flexibility over the quality of ensembles that are generated. The documentation for PEAS and its Google Colab executable platform are located at https://github.com/mitkeng/peas.

**Figure 2:**
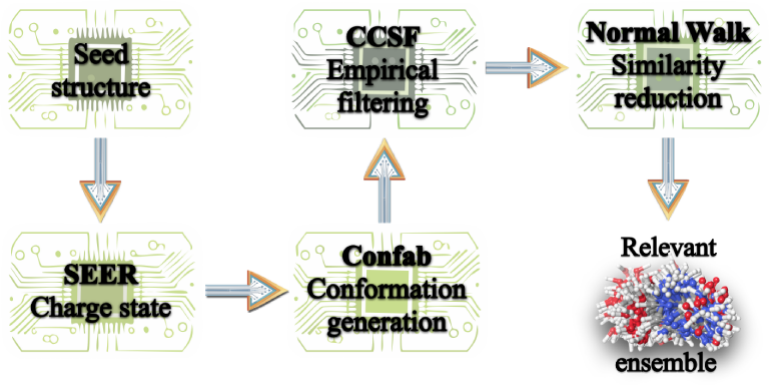
Programmatic workflow showing the three software modules SEER, Confab, and CCSF, integrated seamlessly to obtain an experimentally relevant ensemble.

## Programmatic Details

**The SEER module** initiates the ensemble-generating operation by accepting either a neutral chemical input as a SMILE string or an XYZ format file. Its operation supports the generation of a singly protonated [M + H]^+^ or deprotonated [M – H]^−^ nitrogen or oxygen atom within a given molecule. First, charge site enumeration over all titratable N and O atoms, filtering, and energy ranking are accomplished by the Yggdrasil Decision Forests^19^ Gradient Boosted Trees (GBT) machine learning model. This model was trained on small molecules and biomolecules, with a tripeptide being the largest ion used in the validation set.

Next, the ranked charge models are fed into ANI-2x^20^ for geometry optimization and energy calculation. By default, this step runs on the CPU; however, GPU acceleration can be enabled by changing the runtime settings. Figure 3 shows the computational cost comparison between the CPU and T4 GPU, measured as time per charge model, for 11 diverse test ions. The relative energies calculated for single-point energy values produced by ANI-2x are then used to rerank the charge models. Since ANI-2x energies are computed for optimized and charge site-differentiated geometries, and the GBT energies are obtained from identical geometries (except for the difference in charge site) for charge models, our algorithm ensures that ANI-2x ranking supersedes the initial GBT ranking. The file naming scheme for the results incorporates the rank number into the XYZ output file name. The results are saved in the directory /Completed_Job/[molecule name], where the folder [molecule name] is named after a user-input molecule or system (Figure 4A). Additionally, information pertaining to quantities such as relative energy and gas-phase mole fraction for SEER-assigned equilibrium charge state(s) is reported in a results summary log (i.e., results_sum.log) and saved in the same directory upon run completion.

**Figure 3:**
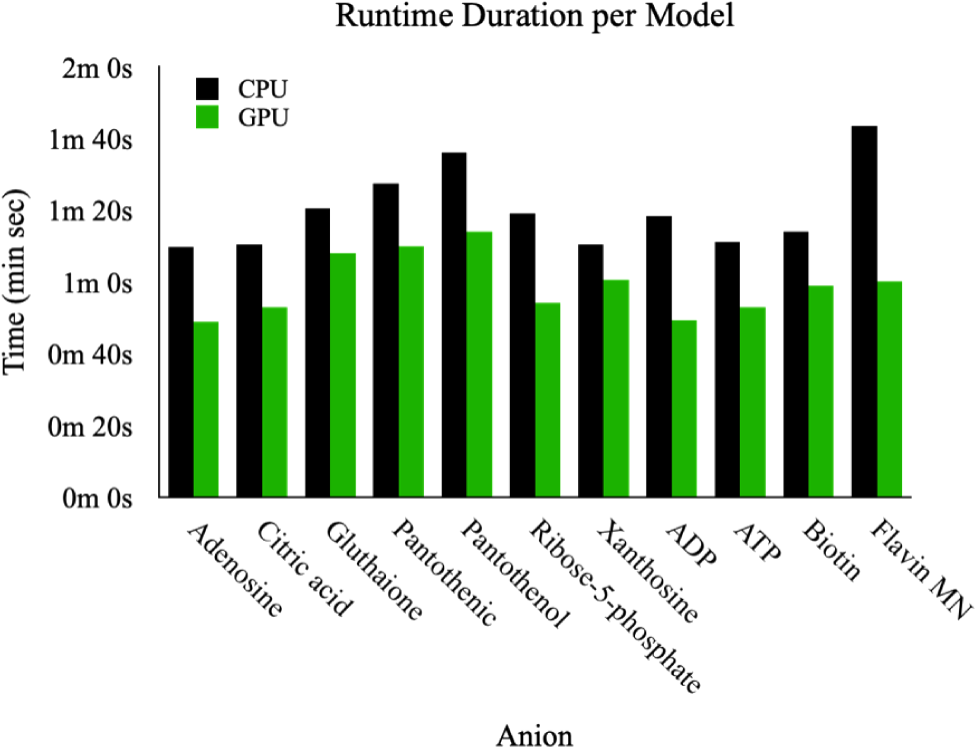
Computational cost evaluated in time per charge model between CPU (AMD EPYC 7B12) and T4 GPU (on a server with Intel­(R) Xeon­(R) CPU @ 2.00 GHz) for the ANI-2x geometry optimization task.

**Figure 4:**
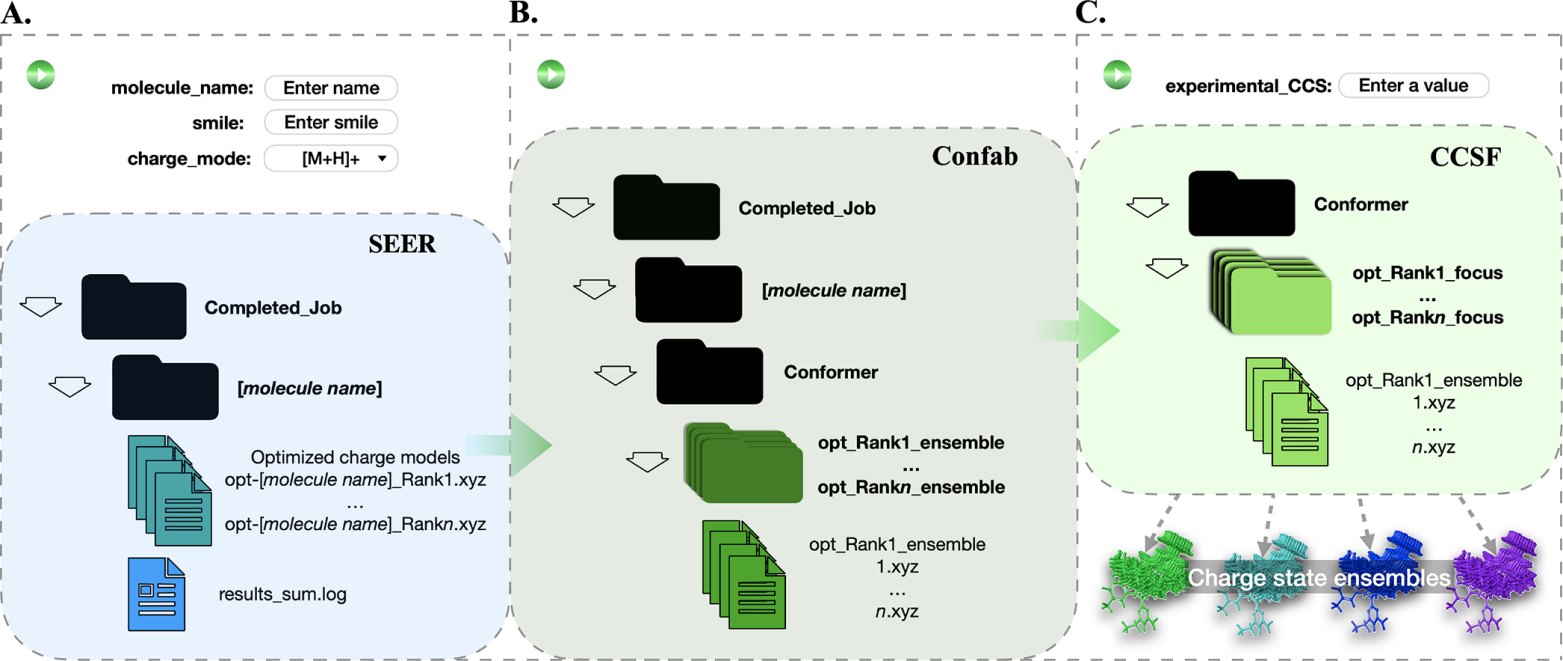
Programmatic inputs and outputs scheme for the three modules A) SEER, B) Confab, and C) CCSF in PEAS. As indicated, each module requires limited or no user input (region above the colored cell) for the program to execute and results to generate. Output files for each module are retained within their respective folders for user reference. The final folder(s) containing the candidate charge state ensemble(s) produced from PEAS (green CCSF module) is automatically converted to a zip file and downloaded upon job completion.

**The Confab module** (Figure 4B) iterates through the ./Completed_Job/[molecule name]/ directory, and for any optimized charge model present, it generates by default a targeted 1000 unique and low-energy conformers. However, depending on the input chemical system, Confab may or may not successfully generate 1000 conformations due to its filter, which removes structurally similar and high-energy conformers. Generally, an insufficient ensemble by our standard (i.e., <1000 conformers) is not an issue since conformers are routinely filtered or screened at a downstream step to remove similarity (this will be described later for PEAS using the normal walk algorithm) for QM processing, as the number of conformers generated is routinely too significant of a workload to be computationally practical. Successfully generated conformers for a charge state model are initially appended to a single file and then subsequently partitioned into individual XYZ files, which are deposited into a charge model ensemble-specified folder in the directory /Completed_Job/[molecule name]/Conformer/opt_Rank­[n]_ensemble/.

**The CCSF module** (Figure 4C) operates using a TensorFlow Keras deep neural network (DNN) model that we trained on features extracted from pre-QM optimized chemical geometries (i.e., raw 3D structures) and DFT-derived CCS values—the labels. Currently, the chemical systems used in the training set include small molecules, lipids, nucleotides, and peptides (ranging from 3 to 17 amino acid residues). Although we used a diverse set of chemical classes, the training examples within each class are limited, with a total of 4,000 combined data points of conformations for all classes.

CCSF takes conformers (i.e., XYZ files) outputted from Confab in the opt_Rank­[n]_ensemble folder and filters them according to their projected experimental viability and, in doing so, distills the overall conformational diversity within an ensemble. CCSF automatically sets filter size is a function of the DNN model training accuracy and a user-defined reference CCS value. What remains after filtering, if the initial conformations are viable, is essentially a conformational space focused on the user reference CCS. Currently, the *R* ^2^ between DFT-derived and CCSF-predicted CCS for all major molecule classes (i.e., lipids, oligopeptides, carbohydrates, and small molecules) is 0.87. The CCSF step is uniquely important because it provides preliminary information on the result quality of a generated ensemble. Accordingly, if none or an insufficient number of reference-relevant conformers are captured, a user can responsively backtrack to the previous step and increase the number of conformers generated by Confab (i.e., num_of conf) to ≫1000. Upon operation completion, any conformer(s) captured by CCSF is transferred to its appropriate rank focus folder located in the directory /Completed_Job/[molecule name]/Conformer/opt_Rank­[n]_focus/. An ensemble that contains fewer than 50 conformers located in this folder is the final job output for PEAS and is downloaded automatically via a zip file upon job completion.

**The normal walk algorithm** is executed in the final step of PEAS to eliminate structures with similar conformations from any remaining ensemble containing more than 50 conformers. This process helps to further reduce the workload later downstream in the computationally costly QM step. Traditionally, we have relied on AutoGraph^21^ to cluster and remove similar conformers; however, to support compatibility with the Python notebook environment, improve scalability, and significantly speed up throughput (10-fold faster), we have elected to use the single value decomposition (SVD) method to evaluate similarity between Cartesian coordinate data points (i.e., XYZ). Normal walk implements SVD to obtain an RMSD similarity score for a tested ensemble. The average and standard deviation of an ensemble RMSD are also calculated and used to establish a normal distribution, whereby the acquired similarity score is compared along this normal distribution, starting from the trailing tail (minimum ensemble RMSD value) to the leading tail (maximum ensemble RMSD value). eq 1 is used to determine the number of steps taken to walk across the normal distribution, where a sampled RMSD (RMSD between the reference conformation and test conformation) is compared to the similarity score.


1 step=20×ROT

where ROT is the number of rotatable bonds for a system and 20 is a heuristically arrived value that supports sufficient sampling of a given normal distribution. Simply put, the number of steps is directly proportional to the number of conformers sampled. Conformers with RMSD values that are less than the ensemble similarity score are removed. The validation and benchmarking for normal walk are addressed in the following section.

## Validation and Benchmarking

**Confab independent validation** is required since this is the first time we are using this generator for gas-phase structure prediction. For a system to have an experimentally valid structure, its equilibrium or energy minimum conformation must achieve a Boltzmann energy (ensemble) weighted CCS error of ≤3% (i.e., CCS agreement between experiment and theory). It should be noted that the 3% error accounts for the uncertainty observed in ion-mobility mass spectrometry-derived CCS values.^22,23^ Upon following the steps for structure assignment (see the blue segment in Figure 1), which implements geometry optimization and single-point energy calculation at the DFT D3BJ-B3LYP/6-31+G­(d,p) and D3BJ-B3LYP/6-31G­(d,p) levels of theory for the [M – H]^−^ and [M + H]^+^ ions, respectively, we obtained an average CCS error of ∼2% for the 10 systems tested (Figure 5). Thus, for 80% of the test systems, Confab is capable of generating experimentally viable structures, which is an acceptable performance.

**Figure 5:**
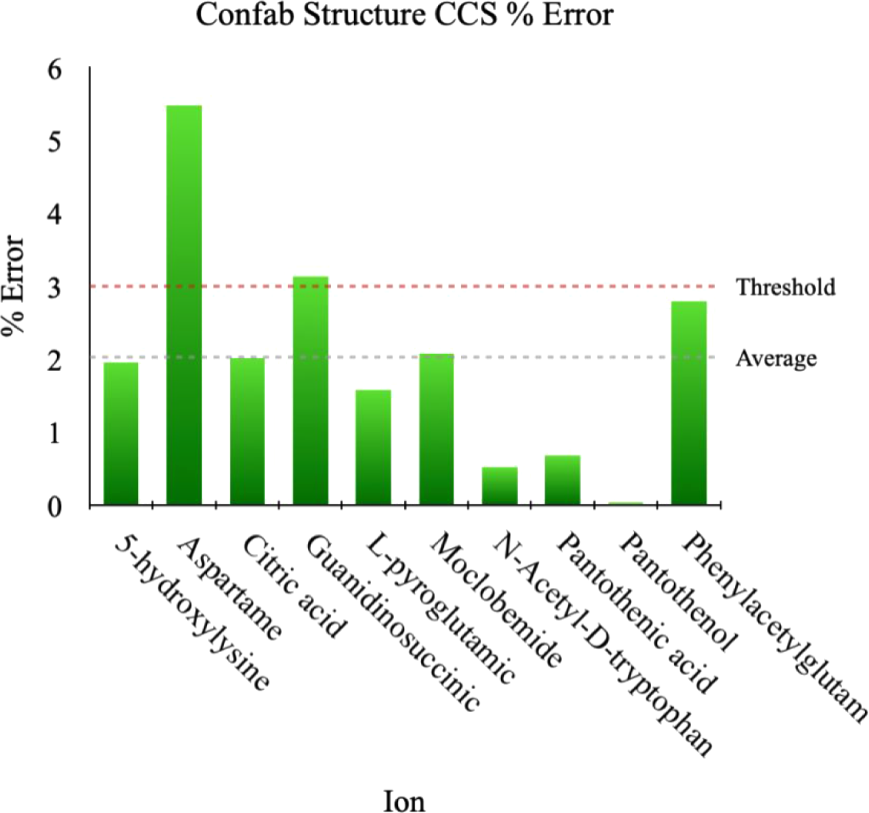
Performance validation study of Confab for generating experimentally viable gas-phase structures. Overall acceptable performance requires achieving an average CCS below the 3% threshold. The average CCS computed for Confab equilibrium structures is ∼2%, with 8 of 10 systems having <3% CCS error. CCS values are Boltzmann-weighted as a function of DFT energy.

**Benchmarking of Confab/CCSF ensemble performance** was assessed by comparing the end results, which was achieved using the standard steps indicated in blue in Figure 1. To benchmark, charge state ensembles for eight test systems generated by Confab/CCSF (PEAS), Confab, or ConfGen were first clustered to eliminate similarity using AutoGraph.^21^ Next, geometry optimization and single-point energy calculations for the clustered ensembles were carried out at DFT D3BJ-B3LYP/6-31+G­(d,p) and D3BJ-B3LYP/6-31G­(d,p) for [M – H]^−^ and [M + H]^+^, respectively. CCS values were then computed for all DFT-optimized conformers using HPCCS.^24^ For Confab/CCSF, Confab, or ConfGen, we assessed both the minimum energy achieved for the predicted equilibrium structure and the corresponding CCS error. Figure 6A shows that for 5 of the 8 test systems, Confab in combination with CCSF (Confab/CCSF) successfully captured the energy minima (mean ΔE ≈ −0.22 kcal/mol), which outperforms either ConfGen or Confab alone. Moreover, the average CCS errors obtained for the energy minimum candidate structures produced are approximately 1.4%, 1.9%, and 1.4% for ConfGen, Confab, and Confab/CCSF (Figure 6B), respectively. Altogether, Confab/CCSF achieves good performance for its intended purpose, with the predicted equilibrium (energy minimum) structures having CCS errors of less than 3%.

**Figure 6:**
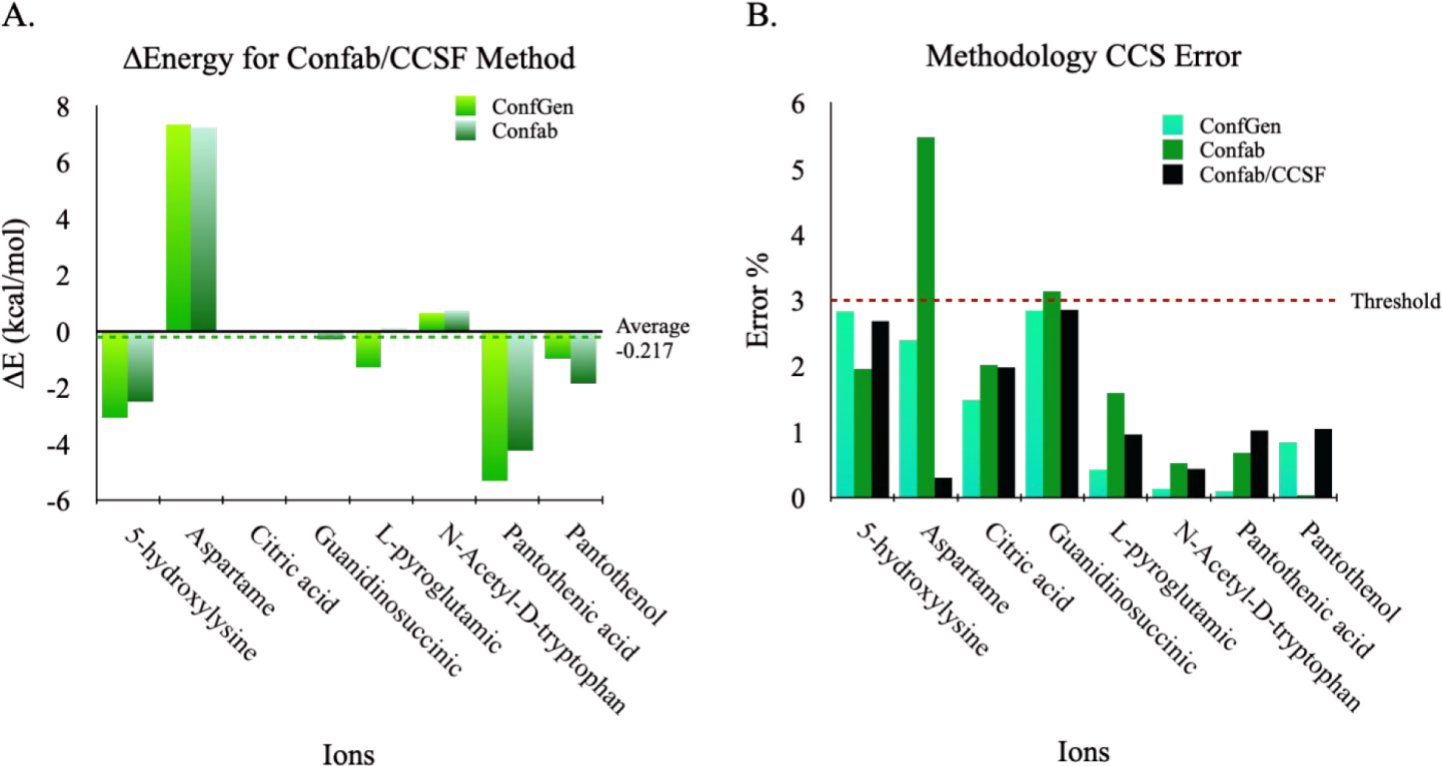
Benchmark results for the PEAS-generated ensemble. The evaluation is for Confab coupled to CCSF (Confab/CCSF) versus ConfGen or Confab alone. All ensembles were then processed using the standard steps to obtain DFT-optimized geometry, DFT single-point energy, and CCS. (A) Comparison of DFT electronic energy for minimum energy structures (equilibrium) produced from PEAS, ConfGen, or Confab. For 5 of 8 systems, PEAS has lower energy than ConfGen or Confab, as seen by the negative ΔE, which signifies that the “global” minimum is captured by PEAS most of the time. (B) Comparison of experimental CCS agreeableness to assess validity of the predicted equilibrium structures. The average CCS errors for ConfGen, Confab, and Confab/CCSF are 1.4%, 1.9%, and 1.4%, respectively.

**Normal walk performance** was benchmarked against AutoGraph by using the ensembles generated from PEAS after the CCSF step. Ideally, we want normal walk to significantly eliminate similarity while retaining enough conformational diversity so that at least one structure is experimentally viable. In general, we found that normal walk resulted in more conformers per ensemble than AutoGraph, and, as expected, the number of conformers increases with an increasing number of rotatable bonds. Results of the predicted CCS in Figure 7A show that normal walk performs as well as AutoGraph. However, the DFT energy spread captured (Figure 7B) by normal walk (mean ≈ 43 kcal/mol) is significantly larger than AutoGraph (mean ≈ 19 kcal/mol), which, for the eight systems in the test set, we feel, is beneficial for locating energy minimum structures. Despite the favorable outcome in energy space, it should be noted that the generally larger ensemble size from normal walk will increase the computational cost downstream, which is due entirely to adding more conformers for QM processing. However, the speed at which it samples prior to the QM step is significantly faster than that of AutoGraph, and the speedup is more dramatic as the ensemble size increases. Also, normal walk can handle a much larger number of conformations in a single run than AutoGraph. For example, any ensemble that contains greater than ∼3000 conformations must be fragmented into smaller ensembles and run separately for AutoGraph to be practical.

**Figure 7:**
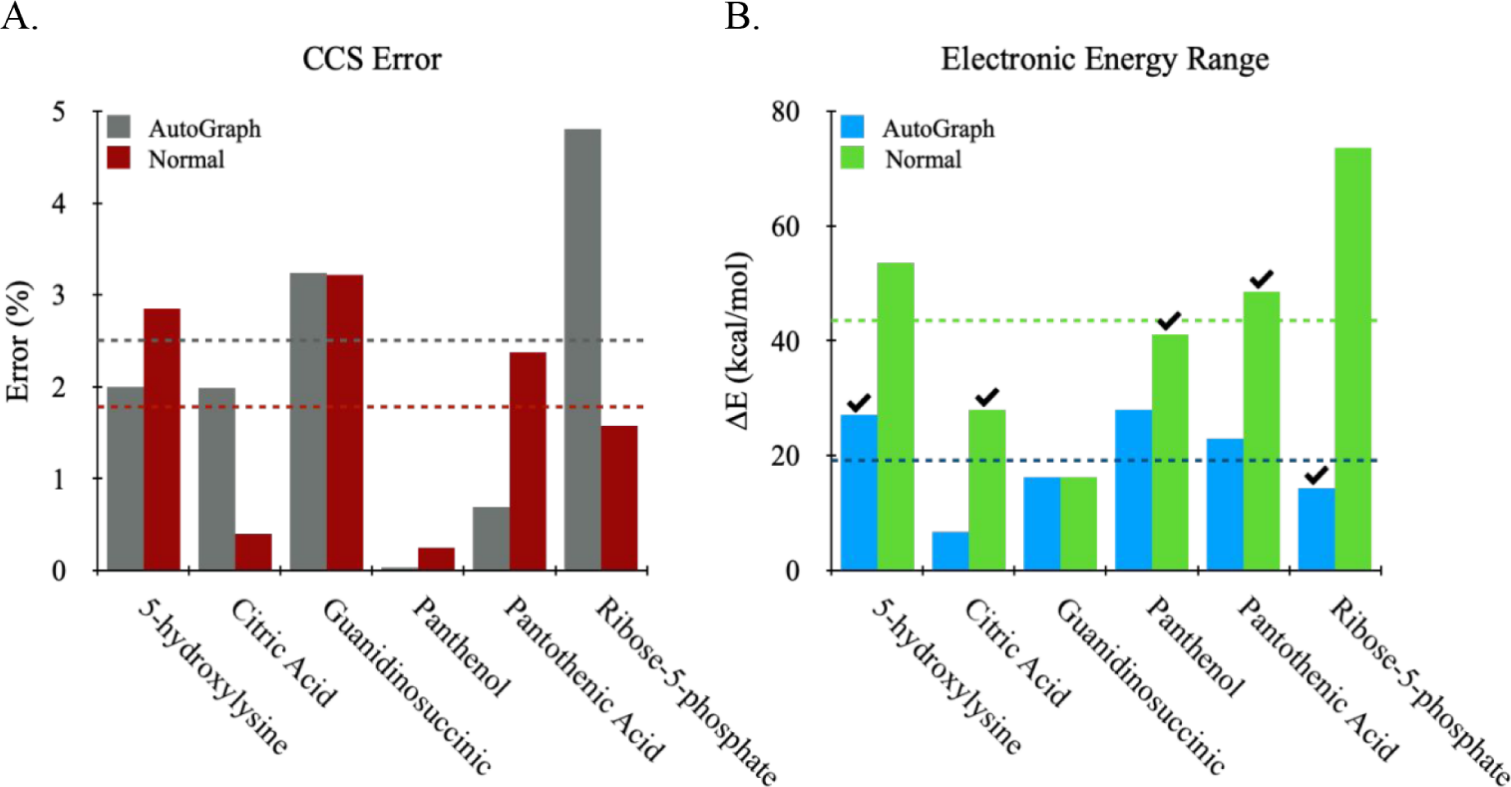
Structure assignment accuracy using the normal walk algorithm compared to AutoGraph for conformer similarity reduction. The eight ions tested have been optimized at the DFT D3BJ-B3LYP/6-31+G­(d,p) or DFT D3BJ-B3LYP/6-31G­(d,p) level of theory. (A) The average predicted CCS errors are ∼2.5% (gray dash) and ∼1.8% (red dash) for AutoGraph and normal walk, respectively. (B) The average DFT energy ranges are ∼19 kcal/mol (blue dash) and ∼43 kcal/mol (green dash) for AutoGraph and normal walk, respectively. The check mark on top of the bar indicates which method captured the energy minimum conformation.

**PEAS whole-program performance** testing was carried out on an additional test set comprising 24 new chemical systems, including diverse small molecules and biomolecules of varying sizes and complexities. Consequently, our results found that for all systems tested, PEAS achieved CCS errors of less than 3%, with a test set average CCS error of 1.63% ± 1 (for complete CCS performance results, see Table S1 in Supporting Information). It should be noted that the reference CCS values used for this trial originated from DTIMS and TWIMS (traveling-wave ion mobility spectrometry). This is acceptable since CCS values derived from DTIMS and TWIMS experiments generally differ by 1–2%.^25^ Thus, we also demonstrate here that PEAS can be applied to capture TWIMS-relevant gas-phase ions.

**PEAS limitations** are directly linked to the limitations of the training sets of the component software packages. For CCSF and SEER, their limitations are addressed in their respective works.^3,9^ As mentioned, PEAS will attempt to generate conformations for [M + H]^+^ or [M – H]^−^ adduct ions for a given user input. Of course, as the size of an input system increases (along with the number of titratable sites), the accuracy of the charge state becomes more difficult to predict. On the other hand, we found that systems with limited titratable sites, like steroid molecules, may not be applicable for PEAS due to the lack of viable protonation or deprotonation sites predicted by our software. In a situation where a protonation or deprotonation site is unsuccessfully assigned, PEAS will terminate with error.

## Conclusion

In this work, we introduced PEAS, a user-friendly Python application for the sampling of IM-MS experimentally relevant gas-phase conformational spaces. We developed PEAS with the specification to include most of the required traditional conformation sampling tasks—which encompass charge state assignment, conformation generation, and relevant conformer screening—streamlined within a single executable run prior to proceeding to quantum mechanical processing. By integrating the component software SEER, Confab, and CCSF into a single platform (i.e., PEAS), we have created an autonomous programmatic workflow that streamlines results by reducing user intervention and file shuffling between different program platforms. Furthermore, we reported that our performance qualification studies (i.e., validation and benchmarking) of PEAS have verified that our software is capable of consistently producing acceptable results within its intended range of application. With that said, we recommend that discretion be exercised when attempting to overextend the PEAS applicability beyond the DTIMS experimental domain or its component software training sets.

## Supplementary Material



## Data Availability

Software documentation and a link to the PEAS web application are accessible at https://github.com/mitkeng/peas.

## Notes

Tabulated test set CCS performance results (PDF)
