# Supplementary material for: PEAS: An Application for Autonomous Precision Conformation Sampling
Source: J Chem Inf Model. 2025 Nov 5;65(22):12161–7. doi: 10.1021/acs.jcim.5c02104 (PMC12648651; doi:10.1021/acs.jcim.5c02104)
Supplement: Supplementary file 1 [file ci5c02104_si_001.pdf]

PEAS: An Application for Autonomous Precision Conformation Sampling

Mithony Keng and Kenneth M Merz, Jr.*

Department of Chemistry, Michigan State University,

East Lansing, Michigan 48824, United States

Department of Biochemistry and Molecular Biology, Michigan State University,

East Lansing, Michigan 48824, United States

*Corresponding Author: Kenneth M. Merz

Table S1. PEAS whole-program CCS performance results for 24 protonated systems. The reference CCS were produced from either DTIMS or TWIMS experiments[1, 2] .

	Ions [M+H]+	CAL CCS (Å^2)	EXP CCS (Å^2)	% Error
**1**	4-aminobenzoic	130.09	131.00	0.69
**2**	Acyclovir	149.30	146.21	2.11
**3**	AICAR	154.53	152.85	1.10
**4**	Alfluzosin	195.94	193.90	1.05
**5**	Althiazide	186.65	181.50	2.84
**6**	Amoxcillin	184.34	187.40	1.63
**7**	Carbidopa	146.71	143.40	2.31
**8**	Cefaclor	167.18	167.90	0.43
**9**	Cilostazol	194.42	199.70	2.64
**10**	Ciprofloxacin	180.30	175.90	2.50
**11**	Cytidine	153.18	152.18	0.66
**12**	DC-MP	161.07	165.59	2.73
**13**	Firocoxib	185.52	181.60	2.16
**14**	Folic acid	196.88	196.20	0.35

**15**	Guanosine	163.84	163.49	0.21
**16**	Karbutilate	168.81	173.50	2.70
**17**	Lenacil	148.76	148.30	0.31
**18**	Mefexamide	167.62	170.53	1.71
**19**	Melatonin	154.92	153.75	0.76
**20**	Omeprazole	183.91	179.67	2.36
**21**	Oxibendazole	164.88	161.40	2.16
**22**	Ribavirin	151.33	149.81	1.01
**23**	Thr-His-Cys	188.19	184.00	2.28
**24**	Tryptophan	146.68	150.30	2.41
	Average			1.63 ±1

REFERENCE

(1) Paglia, G.; Williams, J. P.; Menikarachchi, L.; Thompson, J. W.; Tyldesley-Worster, R.; Halldórsson, S.; Rolfsson, O.; Moseley, A.; Grant, D.; Langridge, J.; et al. Ion Mobility Derived Collision Cross Sections to Support Metabolomics Applications. *Analytical Chemistry* **2014**, *86* (8), 3985-3993. DOI: 10.1021/ac500405x.

(2) Hines, K. M.; Ross, D. H.; Davidson, K. L.; Bush, M. F.; Xu, L. Large-Scale Structural Characterization of Drug and Drug-Like Compounds by High-Throughput Ion Mobility-Mass Spectrometry. *Analytical Chemistry* **2017**, *89* (17), 9023-9030. DOI: 10.1021/acs.analchem.7b01709.
